# Molecular fingerprinting of nanoparticles in complex media with non-contact photoacoustics: beyond the light scattering limit

**DOI:** 10.1038/s41598-018-32580-2

**Published:** 2018-09-26

**Authors:** Ivan Pelivanov, Elena Petrova, Soon Joon Yoon, Zhaoxia Qian, Kathryn Guye, Matthew O’Donnell

**Affiliations:** 1 Department of Bioengineering, University of Washington, Seattle, WA 98195 USA; 2 Department of Chemistry, University of Washington, Seattle, WA 98195 USA

## Abstract

Optical instruments can probe physical systems even to the level of individual molecules. In particular, every molecule, solution, and structure such as a living cell has a unique absorption spectrum representing a molecular fingerprint. This spectrum can help identify a particular molecule from others or quantify its concentration; however, scattering limits molecular fingerprinting within a complex compound and must be overcome. Here, we present a new, non-contact photoacoustic (PA)-based method that can almost completely remove the influence of background light scattering on absorption measurements in heterogeneous highly scattering solutions and, furthermore, separate the intrinsic absorption of nanoscale objects from their scattering. In particular, we measure pure absorption spectra for solutions of gold nanorods (GNRs) as an example of a plasmonic agent and show that these spectra differ from the extinction measured with conventional UV-VIS spectrophotometry. Finally, we show how the original GNR absorption changes when nanoparticles are internalized by cells.

## Introduction

For over 150 years, scientists have used optical extinction/absorption spectroscopy to quantify the molecular constituents of complex media^1,2^. Specifically, ultraviolet through visible light (UV-VIS) spectrophotometry has been a standard laboratory tool since the 1940s for chemical analysis in disciplines ranging from materials science to physical chemistry to molecular biology^3^.

**Figure 1:**
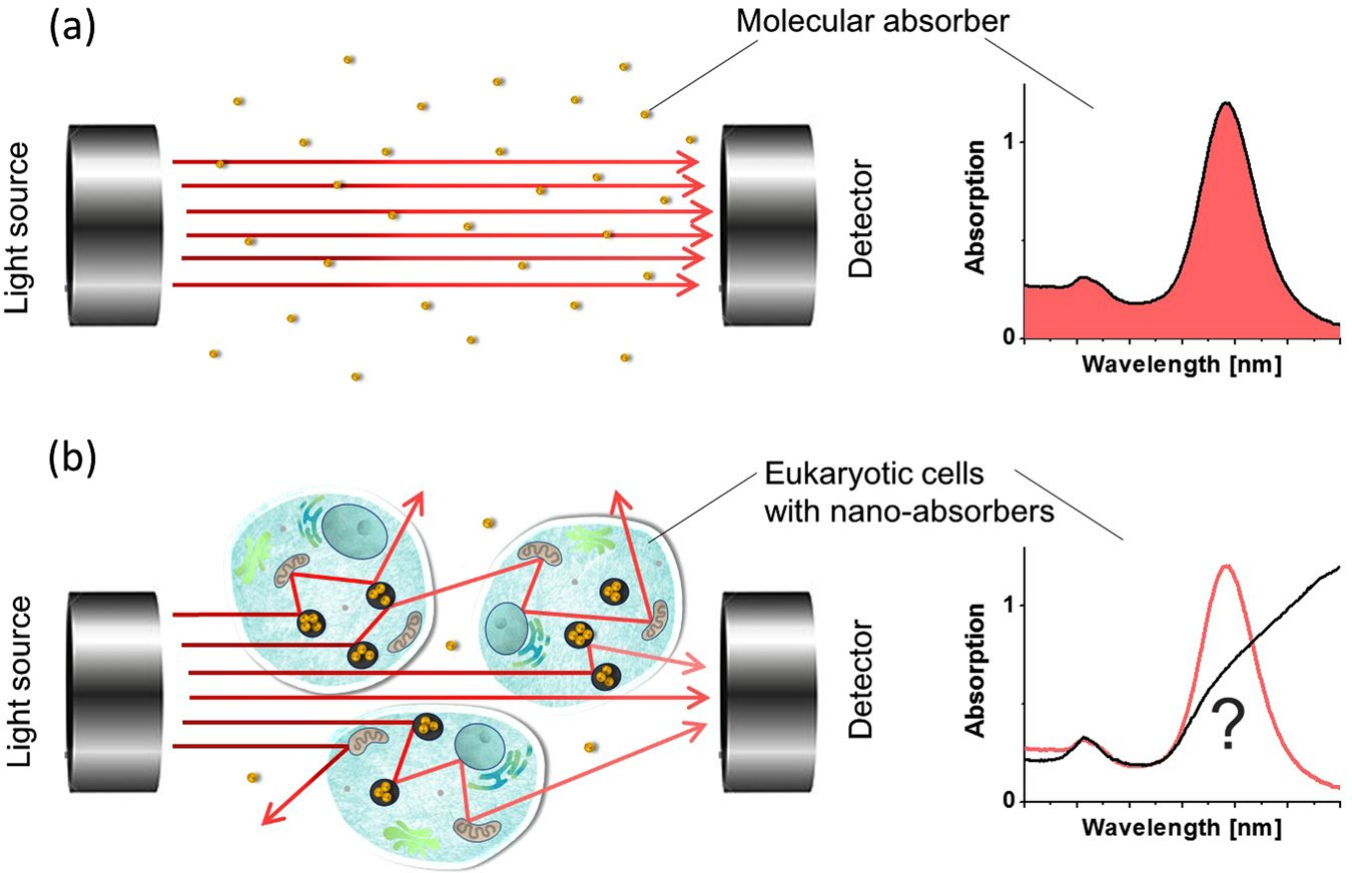
Principle of UV-VIS spectrophotometry. (**a**) Detected UV-VIS spectrum for a pure absorbing material. Light attenuation is produced solely by molecular absorption of probe photons. (**b**) Measurement of the UV-VIS spectrum when part of the incident photon flux is scattered.

In UV-VIS spectrophotometry, the portion of light that does not reach the detector is assumed to be absorbed inside the medium (see Fig. 1a). However, UV-VIS extinction spectra are determined not only by absorption but also by scattering. Differences in refractive indexes of different structures within a complex medium partially reflect light from internal interfaces, thus creating a scattering component. Optical scatterers do not absorb photons, but rather redistribute them, changing their propagation path. Consequently, a considerable fraction of transmitted light does not reach the detector and a conventional spectrophotometry registers incorrect (higher) absorptivity for such media (as shown in Fig. 1b).

Light absorption is an intrinsic property of molecules whereas scattering is an extrinsic property determined by a medium’s detailed structure and morphology. For simple samples such as pure liquids, absorption dominates UV-VIS spectra, providing detailed information about molecular constituents. For more physically complex samples, such as biological tissue, blood, stimulus-responsive materials, suspensions of nanoparticles, polymer films, colloidal and thin film semiconductors, plasmonic nanostructures, metamaterials and others with significant scattering over a broad wavelength range, UV-VIS spectra are hard to interpret and provide few details about molecular constituents. Indeed, this is similar to the simple case of a coin that can be easily seen inside a glass of water, but cannot be detected inside the same glass filled with milk even though milk has absorption very similar to that of water^4,5^.

The problem of extracting light absorption from UV-VIS spectra for media exhibiting a significant scattering background was reported years ago^6–9^, and some pure optical methods have been developed to minimize its effects^6,8,9^. However, these techniques cannot be easily incorporated into conventional spectrometers or multi-spectral devices due to their complexity.

To measure medium absorption and scattering independently, an integrating sphere surrounding the measurement chamber can be used^10^. This approach is appropriate for both homogeneous samples^11^ and nanometer size targets^12^, but is very sensitive to sample size, sample position within the sphere, and the type of chamber. Calibration is required for all wavelengths and full quantitation requires a detailed scattering model inside the sample. In general, it is very difficult to implement for routine measurements in the typical well chambers used by conventional UV-VIS spectrophotometers.

Multiple techniques have also measured the optical properties of biological tissues^13,14^. The most efficient analyzes the propagation time and extension of an ultra-short probe laser pulse due to light scattering in the medium, which if detected, can separate medium absorbance from scattering^14^. However, it requires pico- or femto-second lasers tunable over a wide wavelength range (unavailable on the market) and complicated time-resolved detection techniques. Thus, this approach is not well suited to routine spectrophotometry measurements. In addition, it is not universal, requiring thick, strongly scattering samples and diffusive light propagation to obtain quantitative results.

The situation may be even more complicated. A substance might scatter light itself or combine absorption and scattering properties (like most nanoparticles^15–20^) and, in addition, be within a scattering environment (such as most biological tissues^21^). Obtaining real molecular signatures (or true absorption spectra) becomes extremely complicated for this situation. Indeed, it is extremely difficult to distinguish light absorption from scattering, i.e., find a real molecular fingerprint within a very complex scattering background, which is essential for many applications based on nano- and micro- structures.

Gold nanoparticles (GNPs) are booming in many areas, such as hot electron generation^22^, photovoltaics^23^ and biomedicine, due to their nearly unique optical properties and the ease in conjugating them with important biomolecules^18–20,24–33^. Depending on size, shape, degree of aggregation, and local environment, GNPs exhibit different colors reflecting their plasmonic resonance when irradiated with light of an appropriate wavelength^15–18,20,34–38^. Plasmonic resonance underlies the intense absorption and scattering of light^15,20,34^ and is the basis for many biological sensing and imaging applications.

Molecularly targeted precise drug delivery, immune and nanoassays, gene editing, stem cell surgery and advanced methods of therapy have all leveraged GNP and together are significantly impacting all of biomedicine. Measured NP spectra can drive multi-wavelength schemes to remove background signals (i.e., signals not resulting from the agent) in photoacoustic molecular images^39,40^. Furthermore, for therapy applications (photodynamic or photothermal), UV-VIS spectra are needed to predict the thermal dose at a specific wavelength. Unfortunately, UV-VIS measurements do not accurately predict the absorption spectra needed for these and other applications because plasmonic coupling induces wavelength-dependent light scattering in addition to absorption, as clearly shown in theoretical work^15,41,42^ as a function of GNP material, shape, size and distance between them^15,20,34,41–43^. Unfortunately, such theoretical results cannot generally be used to separate absorption from scattering of measured UV-VIS spectra because synthesized GNPs are not ideal.

Recently, photothermal spectroscopy methods have been developed to measure pure absorption spectra of NPs down to the scale of single particles^44,45^. This approach uses light-to-heat conversion to deflect a probe beam. Because only the absorbed part of light leads to heating, beam deflection is mostly determined by the light absorption coefficient of the medium. However, the method does not take into account changes in laser fluence due to light scattering and thus is limited to very low concentrations, though excellent results have been obtained for individual nanoabsorbers. Photothermal spectroscopy also fails when a strong scattering background surrounds NPs (or any other absorbers).

Having an instrument to measure the true absorption properties of GNP independent of their scattering and the scattering properties of the background would help improve the efficacy of many methods using GNPs and accelerate their translation. Indeed, most current methods identifying GNP state, composition and environment are based on light scattering^18,20,27,30^, which depends on many factors, including not only GNP concentration, but also the micro and macro properties of the background and measurement conditions.

This paper presents a new approach for non-contact photoacoustic (PA) spectrophotometry (see Methods Section) to accurately assess the optical absorption independent of the scattering environment, i.e. it is scattering insensitive even when light scattering is a few orders of magnitude larger than the absorption.

Photoacoustics has been used for a few decades to image absorbing inhomogeneities in highly scattering biological tissues^46–48^. It uses ultrasound transients (PA signals) excited with intensity modulated light and detected with US transducers. Different transducers can be used, including conventional piezoelectric^49,50^ and all-optical^51–54^. However, the absolute majority of optical receivers used for PA imaging require direct coupling with the medium under study, thus limiting their easy integration into UV-VIS devices.

A relatively recent multispectral approach in PA imaging^55,56^ is a very promising method to separate light absorption from the scattering dependent distribution of laser fluence. However, it can only solve the decomposition problem if multiple absorber types are present in the medium with known absorption spectra, but does not solve the problem of absorption and scattering separation when the absorption spectra are unknown.

In this paper, we first consider a simple example of a mixture of a molecular absorber (cupric sulfate) and polystyrene microspheres (scatterers) to demonstrate that the proposed method can carefully assess light absorption of the mixture independent of the concentration of scatterers and their size. Further, we characterize gold nanorods (GNRs) of different size and show that the ratio of absorption/scattering strongly changes with the GNR effective radius and that the proposed PA method can distinguish a true absorption component from the extinction spectrum. Finally, we explore how the pure GNR absorption spectrum changes when cells internalize NPs, and show how PA spectrophotometry can characterize the absorptive properties of these complex nanoparticles in challenging environments.

## Results

### PA measurement of optical absorption in a turbid medium

Even relatively simple samples can produce UV-VIS spectra strongly influenced by scattering. To illustrate this, we measured spectra with a conventional spectrophotometer (Thermo Fisher, model: Evolution 300) in an aqueous solution of a known molecular absorber, cupric sulfate (CuSO_4_*5H_2_O), with different concentrations of polystyrene microspheres (*Cupric sulfate and polystyrene bead microspheres* in Methods Section) of diameter from 0.5 um to 10 um.

**Figure 2:**
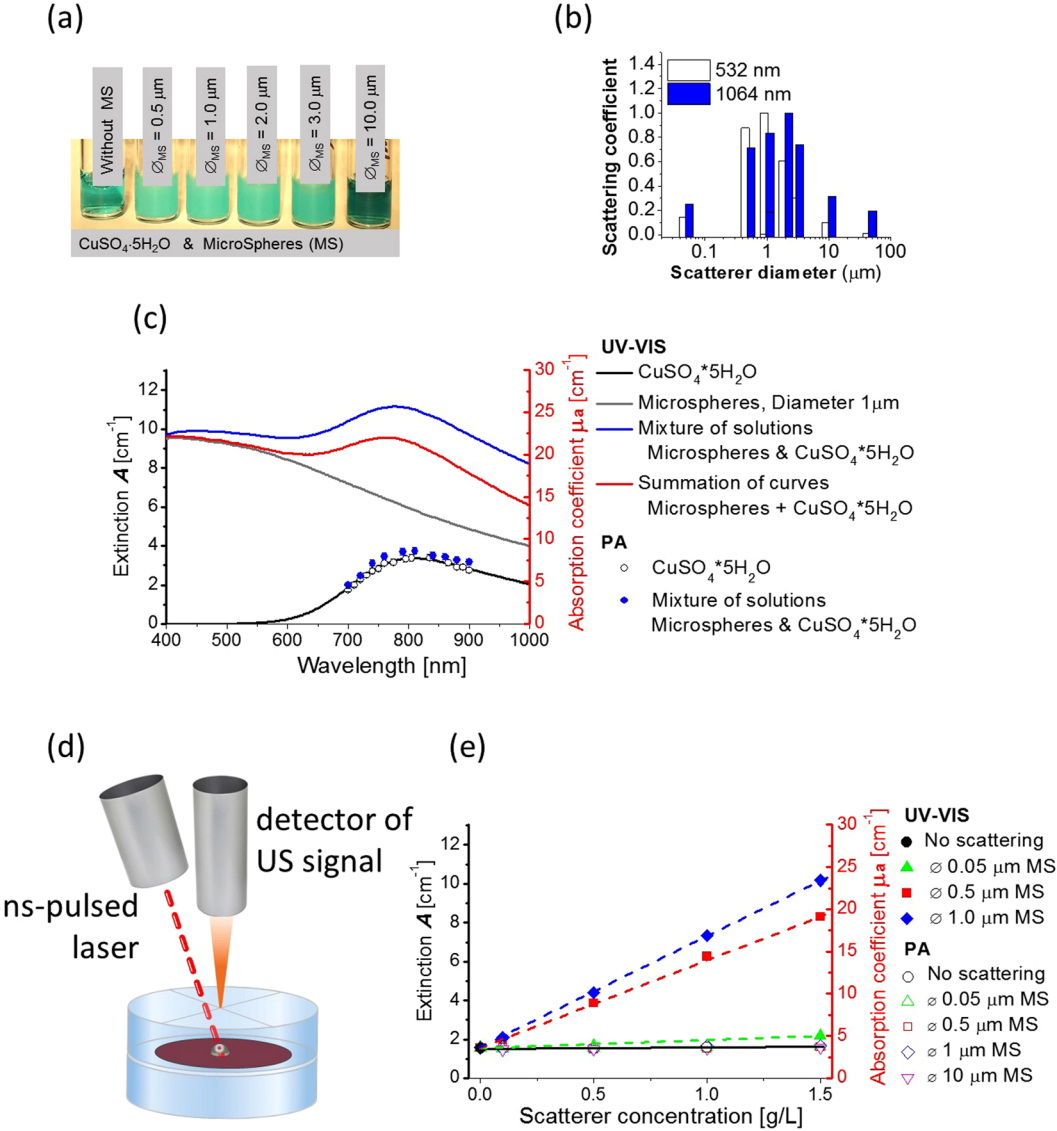
Conventional UV-VIS spectrophotometry versus photoacoustic (PA) spectrophotometry in light scattering solutions with calibrated scatterer size. (**a**) CuSO_4_*5H_2_O solutions (75 g/L concentration) with polystyrene microspheres of different diameters (as labelled) taken at the same concentration (1 g/L) for all microspheres; (**b**) scattering coefficient of an aqueous suspension of microspheres calculated for 532 nm and 1064 nm wavelengths; (**c**) measured spectra of optical extinction with UV-VIS (solid lines) and optical absorption with the PA method (dots) for the CuSO_4_*5H_2_O solution with and without 1 μm diameter microspheres (in concentration of 1.5 g/L). UV-VIS measurements highly overestimate the optical absorption coefficient in the presence of scatterers. Furthermore, summation of the pure absorption spectrum of CuSO_4_*5H_2_O and the scattering spectrum of the aqueous suspension of microspheres (red curve) is very different from the spectrum measured for their mixture (blue curve). PA measurements produce an absorption spectrum of CuSO_4_*5H_2_O nearly identical to the UV-VIS spectrum for the case of no scattering and a slight overestimation for the case of a highly concentrated scattering solution; (**d**) a very simplified measurement diagram for the non-contact photoacoustic (PA) method (see Supplementary Information for details). Photoacoustically-generated ultrasound (US) signals were excited with ns laser pulses emitted from a tunable in (700–900) nm range diode-pumped laser. The sample solution filled a small diaphragm positioned between two quartz plates. US signals were detected from the top surface of the quartz plate with a fiber-optic Sagnac interferometer (see Methods and Supplementary Information for details). The amplitude of the US signal is proportional to the light absorption coefficient and nearly insensitive to light scattering; (**e**) Scatterer concentration dependence of the optical extinction measured with UV-VIS (closed symbols) and optical absorption measured with the PA method (open symbols) at a wavelength of 800 nm. The extinction measured with UV-VIS clearly grows linearly with the concentration of light scatterers, whereas the PA method gives a very stable true absorption measurement even for extremely turbid solutions.

Figure 2a visually compares the turbidity of a CuSO_4_*5H_2_O solution without microspheres (very left vial) to similar suspensions with added microspheres of different diameters (as labelled) at the same volume concentrations. Clearly, they look very different; the turbidity (induced by light scattering) changes with microsphere size and depends on optical wavelength (Fig. 2b). Thus, light scattering can be a very complicated function even for such a simple sample.

Figure 2c (solid lines) presents UV-VIS spectra for an absorbing solution, a scattering suspension, and its mixture (solution of CuSO_4_*5H_2_O with suspended 1 um diameter microspheres). The black curve is the pure CuSO_4_*5H_2_O solution and the gray curve is an aqueous suspension of microspheres. The blue curve presents the measured spectra for a suspension of CuSO_4_*5H_2_O with 1 um diameter microspheres at precisely the same concentrations used to form the black and gray curves. For comparison, the red curve is the summation of black and gray curves, representing a linear combination of UV-VIS spectra for individual components of the simple suspension.

The measured UV-VIS extinction spectrum of the mixture (blue curve – in panel (c)) significantly differs from the sum of absorption and scattering (red curve – in panel (c)). Scattering can greatly modify the UV-VIS spectrum depending on a number of physical parameters such as the relative mechanical densities of the components, size of the scatterers, and their concentrations. Furthermore, the resulting spectrum can change with sample thickness, i.e., the distance between the source and detector, probe beam diameter, and aperture of the detector. Thus, even if scattering within a solution is known, it is often extremely difficult to extract the intrinsic molecular absorption from extinction measurements. This means that molecular decomposition is nearly impossible using UV-VIS spectra obtained in samples with significant optical scattering over the measured wavelength range.

We performed PA measurements on exactly the same medium to produce the results in Fig. 2c. Open black dots correspond to PA measurements on the initial CuSO_4_*5H_2_O solution with no microspheres inside. Clearly, these results match well with the conventional UV-VIS spectrum. PA measurements are limited to the 700–900 nm wavelength range by the current laser, but this range can be easily expanded in the future.

Linearity of the PA signal amplitude versus the concentration of cupric sulfate in solution can be found in Supplementary Information, Supplementary Fig. S1e. A photograph of CuSO_4_*5H_2_O solutions of different concentrations is shown in Supplementary Fig. S2a and PA signal profiles corresponding to these concentrations are plotted in Supplementary Fig. S2b.

Here, and later in this paper, the extinction *A* (typically used in radiometry) is a dimensionless unit defined as

1 $$A=\,\mathrm{log}(I/{I}_{0}),$$

Note, that the extinction *A* measured in a pure absorptive, non-scattering solution is related to the light absorption coefficient defined from the Bouguer-Lambert-Beer law (2):

2 $${\mu }_{a}=\,\mathrm{ln}(I/{I}_{0})/d,(d\,{\rm{is}}\,{\rm{the}}\,{\rm{medium}}\,{\rm{thickness}})$$

for a 1 cm thick cuvette as

3 $$A={\mu }_{a}/\,\mathrm{ln}(10).$$

When the medium scatters light, the Bouguer-Lambert-Beer law (2) no longer applies and the relationship between *A* and ${\mu }_{a}$ is not determined. Scattering redistributes light over the volume of the sample whereas absorption determines attenuation along one direction. Thus, *A* measured in different cuvettes and with different spectrophotometers may differ, whereas ${\mu }_{a}$ always defines the absorbed part of light.

Note also that the extinction measured with a conventional UV-VIS spectrophotometer is not the total light attenuation coefficient (${\mu }_{t}={\mu }_{a}+{\mu }_{s}$)^4,5,57^, and does not equal an effective light attenuation ${\mu }_{eff}=\sqrt{3{\mu }_{a}{\mu }_{s}(1+g)}$ ($g$ is a light scattering anisotropy factor) appropriate for fully diffuse propagation^4,5^. It only determines the ratio of *I* to *I*_0_.

For the proposed method of non-contact PA spectrophotometry (see Methods Section), only the amount of absorbed light is measured and, thus, we can assess how much of ${\mu }_{a}$ (i.e., true absorption) is contained in the extinction *A*.

In Fig. 2, and later figures, we use two scales to readily compare extinction as measured with conventional UV-VIS spectrophotometry and absorption as measured with PA spectrophotometry: the Y axis on the left shows extinction *A* whereas the colored Y axis on the right presents PA measurements and, therefore, displays the light absorption coefficient ${\mu }_{a}\,$determined from (2).

Closed blue dots in Fig. 2c presents the PA absorption measured in the light scattering medium (suspension of CuSO_4_*5H_2_O solution with 1 um polystyrene microspheres of different concentrations). Data very closely match those obtained for the scattering free solution and exhibit the true absorption spectrum of CuSO_4_*5H_2_O, even for a light scattering coefficient close to 10 cm^−1^ with solution turbidity approaching that of milk (a photograph of the solutions can be found in Supplementary Fig. S2c).

Figure 2e shows the measured light absorption as the concentration of light scatterers varies. Conventional UV-VIS outputs continuously growing extinction as a function of scatterer size, whereas the PA measurement always delivers nearly the same value independent of scatterer size and concentration (see Supplementary Fig. S2d for more details). This result clearly shows that properly designed PA measurements with a non-contact system can accurately probe true absorption spectra in highly scattering media without knowing any details about the scattering background.

### Absorption spectrum of GNRs

Conventional UV-VIS spectrophotometry has been used extensively to characterize the optical properties of plasmonic nanoparticles used in biomedical diagnostic and therapeutic procedures^15,17,18,20,26,34–38^. Unlike the simple two-component solution presented above, however, plasmonic nanoparticles both scatter and absorb light efficiently. This makes it exceedingly difficult to separate these components from UV-VIS spectrophotometry measurements alone.

**Figure 3:**
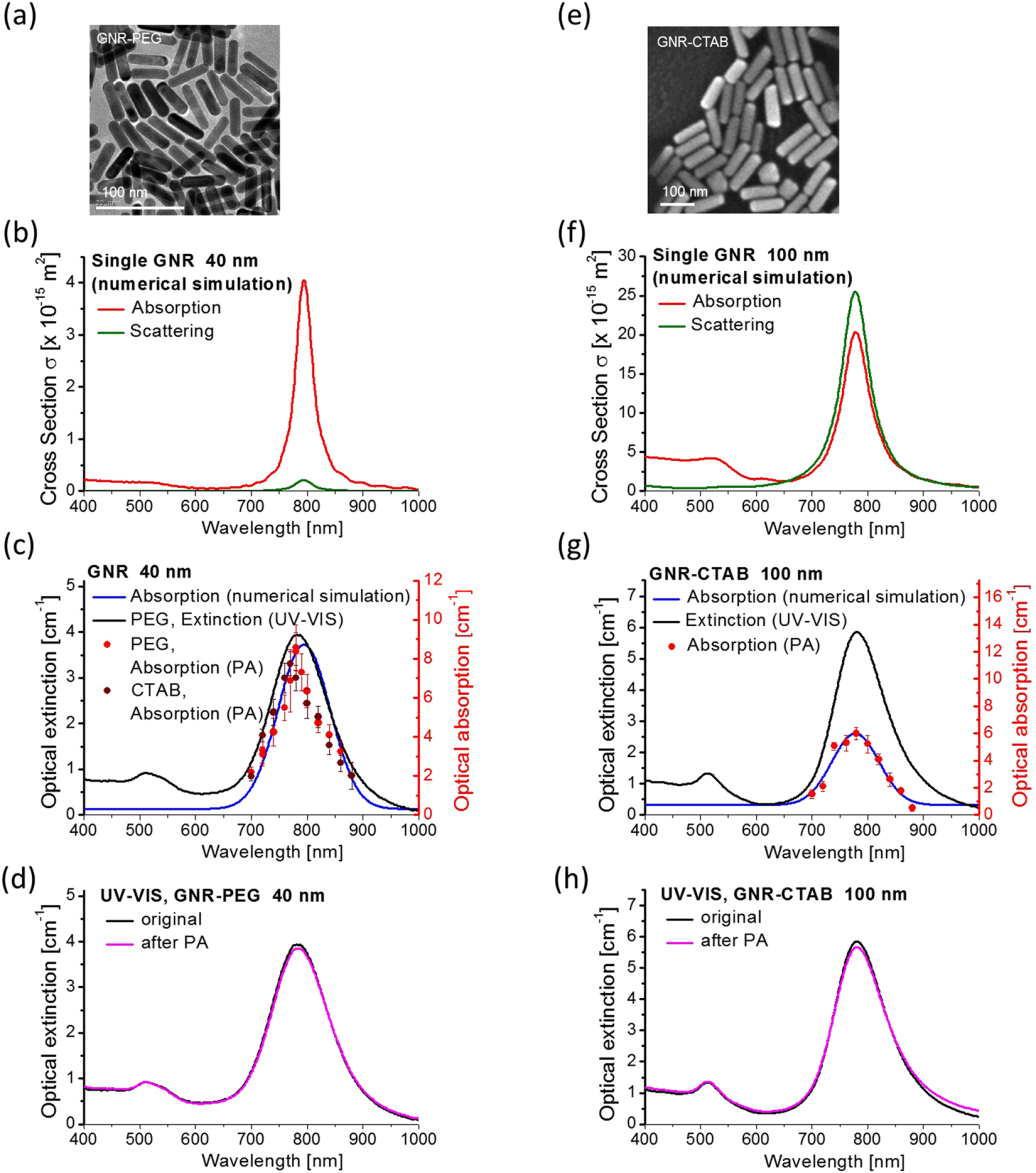
Absorption, scattering and extinction spectra of GNR solutions: numerical simulation, non-contact photoacoustic spectrophotometry, and conventional UV-VIS spectrophotometry. Gold nanorods (GNRs) of different length (around 40 nm and around 100 nm) but similar aspect ratio (the ratio of diameter to length) were used (see Methods for details). Panels (**a,e**), respectively, show their TEM images. Numerically simulated absorption and scattering spectra of a single gold nanorod: panels (**b,f**) for 40 nm and 100 nm, respectively. Comparison of optical absorption spectra for solutions of standard CTAB and PEG coated GNR obtained with numerical simulation, PA spectrophotometry, and UV-VIS spectrophotometry: panels (**c,g**) for 40 nm and 100 nm, respectively. Extinction spectra of GNR solutions (panels (**d,h**) for 40 nm and 100 nm, respectively) measured before and after PA measurements.

Solutions of different sized GNRs (see Fig. 3a,e and Methods Section for details) but with close aspect ratios producing an absorption maximum for the longitudinal resonance near 800 nm (center of the laser range) were studied. For both UV-VIS and PA measurements, aqueous solutions of GNRs were formed with different concentrations to maintain extinction in the range of ($A\cong 1-\,5$) for all samples measured in a 1 cm thick cuvette. Note that for all measurements, we used CTAB and PEG coated GNRs (as indicated in the caption to Fig. 3).

Numerical FDTD simulations were performed using the Lumerical Solutions, Inc. package (version 8.19.1466) to find absorption, scattering and extinction spectra of experimentally used GNR solutions (see Methods Section for details).

Figure 3b, f plot simulated spectra for a single gold nanorod showing that the relationship between absorption and scattering spectral components dramatically changes with GNR size. For small (~40 nm) GNR, the scattering component of the spectrum is less than 10% of the absorption, but for bigger ones (~100 nm) scattering is already higher than absorption.

Figure 3c,g compare numerically simulated optical absorption for an ensemble of GNR (accounting for their size distribution), the extinction *A* measured in GNR solutions using conventional UV-VIS spectrophotometry, and the absorption coefficient ${\mu }_{a}$ measured in GNR solutions with PA spectrophotometry. As seen, PA measurements confirm theoretical predictions very well whereas conventional UV-VIS spectrophotometry gives very different results. The difference is especially clear for larger GNR. Note again that the extinction, *A*, measured with conventional UV-VIS spectrophotometry is not generally the sum of absorption and scattering^4,5,57^.

The fact that absorption is a much smaller fraction of the total extinction with increasing GNR size can have simple practical consequences. For example, by interpreting UV-VIS spectra as reflecting primarily absorption characteristics, photothermal therapies tuned to the wavelength of the extinction peak will produce heating patterns much smaller than predicted for larger GNRs.

We measured solutions with different concentrations and all of them gave the same value of ${\mu }_{a}$. We also measured UV-VIS spectra before and after PA measurements. Results of all of these studies strongly suggest that our PA measurements in the range of laser fluences used (~0.5 mJ/cm^2^) do not change GNR shape and function, at least at the radiation dose received from up to 5000 laser shots at the concentrations investigated. Figure 3d,h show nearly no changes in GNR extinction spectra before and after PA measurements.

For all measurements in an aqueous environment, we assume that Г, the efficiency of PA excitation, is constant and equal to the value for water. For aqueous solutions with a low concentration of biological substances, this approximation is accurate within 10–15%^58^. We also calculated the accuracy of the calibration based on a cupric sulfate solution as ~2% based on the concentration used and studies performed earlier for similar solutions^59^.

However, when metal NP are used, it may seem that this approximation is no longer valid. In fact, NPs themselves do not produce the PA signal; they absorb light and are directly heated, but due to their very high thermal conductivity, heat is released into the surrounding medium and, thus, a “coat” surrounding the NP produces the major PA signal. Multiple papers describe this phenomenon^58,60–62^.

In general, the parameter Г should be known for the substance under investigation. It can be calculated or measured separately with, for example, the method discussed in^59^.

### Absorption spectrum of GNRs internalized by cells

Next, we look at the more complicated problem of measuring the absorption spectrum of nanoparticles internalized by cells. This is a complex optical environment in which nanoparticles themselves scatter significant light (similar to the case presented in Fig. 3), and cellular structures also produce significant background scatter (similar to the case presented in Fig. 2) that can confound optical estimates of the absorption spectrum.

A shift of the extinction peak for internalized GNP was shown in multiple studies^20,29,39^ based on conventional UV-VIS measurements, but no clear methods or instruments were used to quantify pure light absorption. Endocytosis and intracellular trafficking of nanoparticles can create clusters leading to plasmonic coupling between particles^20,29,39,63,64^. The specific type of coupling can depend on GNP’s pathway within cells^63,64^. To ensure successful absorption-based procedures with minimal side effects, the absorption spectra of GNP internalized by cells must be measured. Conventional UV-VIS spectrophotometry cannot separate optical absorption from scattering and, therefore, cannot provide an accurate measurement of light absorption in a simple aqueous environment (see Figs 2 and 3).

Quantifying GNP absorption within cells or within other tissues is quite complicated because light scattering is not only intrinsic (provided by the particles themselves), but also is induced by high scattering cellular components. Separating them is extremely difficult with a pure optical approach. A solution could have significant impact on the design and optimization of imaging and therapy procedures exploiting the optical characteristics of plasmonic nanoparticles.

To study changes in the optical properties of nanoparticles internalized by cells, HUVEC cells and PEG coated 40 nm GNRs were used. The procedure for cell culturing and particle uptake is described in Methods.

**Figure 4:**
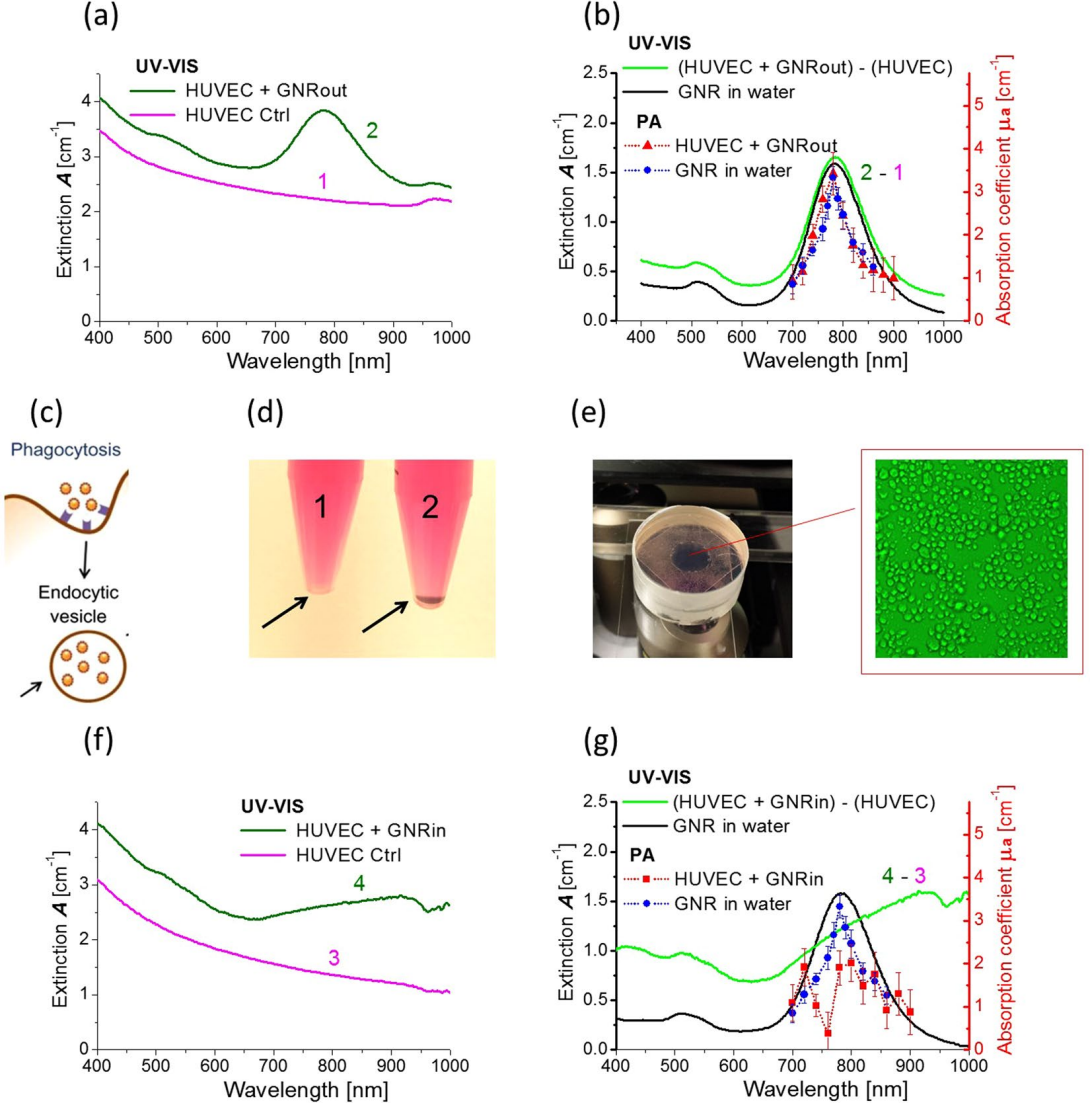
Non-contact PA spectrophotometry of GNR uptake by HUVEC cells. (**a**) UV-VIS spectra of HUVEC cell solution in water (magenta curve, ***1***) and a mixture of HUVEC cells and GNRs (dark green curve, ***2***). GNRs are positioned outside of cells in the mixture; (**b**) subtraction of curves ***2*** and ***1*** (light green curve), UV-VIS spectrum of GNR solution in water (black curve), PA measurements in GNR solution (blue dots) and in the mixture of HUVEC cells and GNRs (red triangles); (**c**) Schematic of cellular uptake of GNRs (replicated from^64^); (**d**) photograph of the reference solution of cells (1) and cells loaded with GNRs (2); (**e**) a cuvette for PA measurements with a solution of HUVEC cells with internalized GNRs; an optical microscope image in the insert shows the approximate concentration of HUVEC cells; (**f**) UV-VIS spectra of HUVEC control solution (magenta curve - ***3***) and HUVEC cells with internalized GNRs (dark green curve - ***4***); (**g**) subtraction of curves ***4*** and ***3*** (light green curve), UV-VIS spectrum of GNR solution in water (black curve), PA measurements in GNR solution (blue dots) and in the solution of HUVEC cells with internalized GNRs (red rectangles).

First, UV-VIS and PA measurements were performed for a solution of cells and GNRs immediately after nanoparticles had been added. Thus, measurements were made before GNRs internalized. We call this solution “HUVEC + GNRout”, i.e., GNRs are outside the cells. The extinction spectrum for a solution of cells without any GNRs is shown in the magenta curve (curve 1) in Fig. 4a to characterize the background scattering environment. The dark green curve (curve 2) in Fig. 4d illustrates the spectrum of the mixture HUVEC + GNRout, which indicates an absorption peak near the maximum for GNRs alone.

Although maximum light extinction is very near the same wavelength, a simple subtraction of curves 2 and 1 (see light green curve in Fig. 4b) does not match the extinction spectrum of GNRs alone. This result clearly indicates the same problem demonstrated in Fig. 2, namely that scattering and absorption properties are not additive in conventional UV-VIS measurements and extracting the true absorption spectrum cannot be obtained simply by subtracting the spectrum measured for a control medium from the total extinction spectrum. In addition, having such a reference measurement is not always possible.

Here we again show the power of PA spectrophotometry: the measured absorption spectra for the GNR solution and for the mixture of HUVEC + GNRout are nearly identical. This is entirely reasonable because GNRs do not change their shape nor significantly cluster in the mixture.

The final experiment was performed for GNRs internalized by HUVEC, i.e., inside cells (Fig. 4c–g). Figure 4d presents a clear visual difference between HUVEC cells alone in aqueous solution and cells loaded with GNRs. UV-VIS spectra of the control HUVEC cell solution and HUVEC-GNRin solutions are presented in Fig. 4f. Clearly, the cells induced a dramatic change in extinction. However, the spectrum looks very different from that for nanorods alone in both UV-VIS and PA measurements. The reason is that nanorods cluster, presumably due to endosomal capture, with internal plasmonic coupling modifying the extinction spectrum, similar to the results in ref.^42^. The main question is which part of this spectrum corresponds to light absorption and which part results from light scattering by both cells and GNR clusters.

To answer this question, the absorption spectrum of the HUVEC-GNRin solution was measured with PA spectrophotometry, as shown by the red curve in Fig. 4g. The measured spectrum is very different from that predicted by conventional UV-VIS spectrophotometry. It is highly reproducible and cannot be explained by measurement error.

Plasmon coupling of a pair of similar aspect ratio nanorods assembled in a nonparallel orientation can explain the unexpected reduction and splitting of the GNR absorption maximum^41,42,65^. While the end-to-end (angle 0°) linkage leads to a red-shift of the longitudinal plasmon band, side-by-side (angle 90°) assembly produces a blue-shift of the absorption maximum. The nonparallel (angle about 45°) assembly of nanorods results in a coupled plasmon resonance with both blue-shifted and red-shifted components. Such inter-nanorod plasmon coupling significantly reduces the absorption maximum. We hypothesize that there may be a mixture of GNR coupling at different angles within the cells of this preparation.

## Discussion

In this paper, we have described a new non-contact PA-based method of optical absorption spectrophotometry that can almost completely remove the influence of light scattering on absorption measurements in heterogeneous highly scattering solutions and, furthermore, separate the intrinsic absorption of nanoscale objects from their scattering. Because the proposed method operates in reflection mode, it is not limited by the concentration of absorbers and scatterers and, therefore, can probe optically dense solutions.

Numerous studies have proposed using nanoparticles targeted to specific cell types for different medical applications, such as sensing, drug delivery, imaging, and therapy. Plasmonic coupling of metallic particles can change the spectrum and sometimes provides highly efficient light absorption and scattering at a specific wavelength, but at the same time, plasmonic properties depend greatly on their local environment as well as their composition.

The nearly unique optical properties of GNP can help greatly improve biochemical assays such as Sandwich-Type Enzyme Linked Immunosorbant Assay (ELISA). It is the most widely used immunoassay to detect a specific antigen within a complex mixture^66,67^. Although very effective at detecting small amounts of analyte, conventional ELISA relies on either fluorescence or a colorimetric change to determine antigen concentrations, requiring purified, cell-free specimens and multiple rinsing steps to minimize optical interference and obtain a pure signal^66^. ELISA’s dependence on enzymatic activity for detection is another problem, due to its variability with temperature, denaturation, pH, and other factors. GNPs can potentially solve these issues^68^, but antibody conjugated particles exposed to an analyte tend to cluster. Clustering alters both the scattering characteristics and absorption spectra of intracellular nanoparticles, where these changes depend greatly on the details of clustering mechanisms within specific cell types. Since optical scattering is a three-dimensional effect strongly dependent on scatterer concentration, true quantitation is challenging. Similar conclusions apply to other scattering-based modalities for sensing and imaging. Even relatively new absorption-based PA imaging may give confusing results when spectral changes of GNP were not quantitatively determined before imaging. Establishing the true absorption spectrum is also extremely important for GNP-based therapies (photothermal and photodynamic).

In this paper, we have demonstrated several very important examples where quantifying the true light absorption spectrum can have significant impact. We have shown that NP (GNRs, as an example) extinction spectrum can be very different from true absorption due to a significant scattering component (as seen in Fig. 3) that increases dramatically with NP size.

Moreover, a NP’s original absorption spectrum can dramatically change when internalized by cells (see Fig. 4). Because the plasmon resonance is very sensitive to geometry of the resonance oscillator and its surrounding environment, any alterations in a NP’s absorption spectrum may provide precise feedback on its state within a cell. A simple absorption-based, label-free technique has many advantages over scattering-based methods.

Because spectral shifts for internalized GNRs dependent greatly on the details of nanoparticle size, concentration, and arrangement within cells, the results in Fig. 4 are specific to the model system used here. Future studies must explore how spectral changes correlate with these parameters for different types of GNP and different cell types. Nevertheless, we believe that this example is very important because it shows that non-contact, PA spectrophotometry represents a new tool for the microscale with the potential for molecular decomposition based on measurements of the true optical absorption spectrum in complex media.

Scattering can lead to serious mistakes in evaluating light absorption with conventional UV-VIS spectrophotometry. Although the scattering component of the spectrum can be estimated using numerical simulations for both a single NP and aqueous solutions, it is extremely difficult for practical cases where NPs interact with living objects such as cells. Numerical calculation of the absorption must account for particles’ interaction with each other, e.g. clustering, and on their interaction with cells. Because these processes depend on numerous parameters, accurate physical modeling is a very complicated, multi-parameter problem. Direct measurement of the absorption spectrum is much simpler and potentially more accurate.

We hope that the technique presented here can immediately be applied in several areas. PA-based absorption spectroscopy may be a good tool to probe any scattering medium, especially biological substances, cell cultures, and tissue samples. We believe that sensing, imaging and therapy (like photodynamic or photothermal ones) applications can only be optimized by accurately measuring a contrast agent’s properties *in situ* to define the best conditions (e.g., wavelength) for optical delivery.

Measurement of true optical absorption of NP-based contrast agents combined with numerical decomposition algorithms may potentially simplify sensing methods and create new absorption-based nanoassays. For example, it might be possible to track the “journey” of a NP within a cell from the measured spectrum at its final “destination” with no need for fluorescent probes that complicate NP-based agents, potentially reducing their delivery to a target and modifying their function. A similar approach can be applied in NP-targeted drug delivery.

The proposed PA approach can be extended to imaging, histology and cytometry. It is not limited to nanoparticles or cells and can be broadly applied in analytic chemistry, especially to monitor fast changing dynamic processes and to characterize complex structures, including ones used in the solar industry.

Finally, we believe that the capabilities of conventional UV-VIS spectrophotometry can also be greatly extended by integrating it with non-contact PA spectrophotometry into a single device.

## Methods

### Non-contact photoacoustic spectrophotometry

The proposed method exploits the photoacoustic (PA) effect^46^, in which a portion of time-modulated light energy absorbed in a target is converted into acoustic transients, i.e., ultrasound (US) signals, acting as a vehicle delivering molecular fingerprints outside of the target where they can be detected.

The PA signal amplitude *P* is proportional to the amount of heat converted into mechanical motion and, thus, proportional to the light absorption coefficient ${\mu }_{a}$^46,69^:

4 $$P={\mu }_{a}\cdot {\rm{\Gamma }}\cdot F({\mu }_{a},{\mu }_{s},g)$$

Here ${\rm{\Gamma }}$ is the efficiency of PA excitation and $F({\mu }_{a},\,\,{\mu }_{s}\,,\,g)$ is the laser fluence in the medium under study as a function of light absorption ${\mu }_{a}$, light scattering $\,{\mu }_{s}\,$, and anisotropy factor $g$.

The PA method was first applied (to our knowledge) to accurately measure the light absorption coefficient in homogeneously absorbing solutions in^69^, which was then extended to strongly scattering (or turbid) media^4,5,70–72^. Recently, the method was proposed and applied in analytic chemistry to analyze optically dense solutions^73,74^.

In spite of the high potential for PA methods in complex optical environments, and extensive work in PA spectroscopy and imaging, a non-contact method for spectrophotometry accurately measuring UV-VIS absorption spectra of arbitrary solutions has not been reported to date.

In this paper, we present a new approach for non-contact PA spectrophotometry to accurately assess optical absorption independent of the scattering environment, i.e. it is scattering insensitive even when light scattering is a few orders of magnitude larger than the absorption. In the proposed method, PA signals are excited in the subsurface layer of the medium under study (see Fig. 2d). A diode-pumped nanosecond laser (Laser-export ltd., model: TiSon-B) tunable over the wavelength range of (700–900) nm was used for PA signal excitation. It has a nearly unique feature of extremely fast (switching time less than one ms) wavelength switching and high (1 kHz and higher) pulse repetition rates, making the PA spectrophotometry fast enough for routine practical use. To avoid local overheating of highly absorptive contrast agents and to avoid thermal non-linearity effects on the PA excitation process^60,61^, the initial pulse energy was attenuated with neutral-density filters (calibrated in the measurement wavelength range) and varied from 1–2 *μ*J depending on the wavelength.

Laser radiation was then focused to a spot size of about 0.5 mm within the medium (solution) under study placed between two non-absorbing quartz plates in a diaphragm 0.1 mm thick and 6 mm in inner diameter. A “narrow” optical beam (see refs^71,72^ and Supplementary Information for details) nearly eliminates the dependence of laser fluence on optical properties, thus making the PA signal linearly dependent on optical absorption. This condition applies when the diameter of the excitation beam is less than the transport mean free path of a photon in the medium^4,5,57^. This means that the PA signal amplitude is nearly independent of light scattering for a 0.5 mm diameter beam in media where the scattering coefficient ranges from 0–20 cm^−1^.

Conventional UV-VIS spectrophotometry does not require mechanical contact to a sample and is very convenient to use, whereas conventional PA measurements require direct acoustic coupling to either piezoelectric or optical transducers. This disadvantage certainly limits potential applications and makes it difficult to combine with conventional UV-VIS instruments into a single device. To overcome this limitation, we also present here a non-contact approach to detect US signals from the front surface of the measurement chamber (having a reflective coating tuned for 1550 nm) with a low-power probe optical beam (see Fig. 2d) acting as part of a remote, ultrasensitive fiber optic Sagnac interferometer (see ref.^75^ and Supplementary Information for details). Another advantage is that it is point-like, i.e. lateral resolution is not determined by the acoustic diffraction limit^75^ but by the optical probe beam spot size, which can be tens of micrometers and less. Thus, unlike conventional photoacoustic microscopy (PAM) systems, both excitation of the PA signal and its detection are optical in the method proposed here (see Supplementary Information, Supplementary Fig. S1 for more details).

To homogeneously probe the medium and average possible sample heterogeneities over the entire medium volume and reduce sedimentation of cells, the measurement cuvette was placed on a XY translation stage (see Supplementary Fig. S1). Motion was position synchronized with laser firing, providing a trigger signal to the laser for every 0.5 mm of translation at a speed of 80 mm/s. Overall, five thousand (5000) laser shots were used for every wavelength in the (700–900) nm range during measurements lasting around 30 seconds per wavelength. Spectroscopic measurements were performed with a step of 10 nm, resulting in 21 points in total over a complete experiment period of 10.5 minutes.

Recorded PA signals were digitized with a 14 bit ADC and then processed with a PC to obtain final absorption spectra.

To measure the absolute value of light absorption, the system was calibrated to a medium with known absorption coefficient, as described in Supplementary Information. Supplementary Fig. S1e shows the linearity of the PA signal over a very broad range of concentrations. Optical absorption spectra were measured for a pure absorptive (scattering free) molecular solution (CuSO_4_*5H_2_O) with the conventional UV-VIS technique and with the proposed PA method. Supplementary Fig. S1f compares measurements to determine the absolute sensitivity of the PA spectrophotometer, e.g. the relationship between the amplitude of the PA signal measured in Volts to light absorption coefficient μ_a_ in cm^−1^.

### Cupric sulfate and polystyrene bead microspheres

Milli-Q water was used for solution preparation. Pentahydrate cupric sulfate CuSO_4_·5H_2_O of 99% purity was purchased from Macron. A highly concentrated aqueous solution of CuSO_4_·5H_2_O was used to prepare mixtures with polystyrene bead microspheres at the final 150 mg/mL CuSO_4_·5H_2_O concentration. The polystyrene (PS) beads microsphere kit (Polisciences, catalog no.19822–1) contained vials with 2.6% suspensions of 0.5 µm, 0.75 µm, 1.0 µm, 2.0 µm, 3.0 µm diameter particles; separately purchased vials contained PS beads of diameters 0.05 µm and 10.0 µm. Precipitation of the microspheres in CuSO_4_·5H_2_O aqueous solution was very slow. Separation occurred 6–8 hours after shaking and resulted in either precipitation or floating depending on the density of the liquid environment.

UV-VIS spectra of solutions were measured by the spectrophotometer (Thermo Fisher, model: Evolution 300) using plastic 1 cm pathlength cuvettes and/or quartz 0.5 cm and 0.1 cm pathlength cuvettes.

### GNR solution

Gold nanorods of about 40 nm and about 100 nm length were purchased from Nanohybrids (Austin, TX).

Two types of 40 nm GNRs were used: PEG and CTAB coated. The localized surface plasmon resonance (LSPR) of CTAB-coated GNRs had the longitudinal peak at 773 nm with an 80% width of 61 nm. The transverse peak was at 512 nm, and the ratio of longitudinal to transverse optical density (OD) was 1.09/0.25 = 4.36. GNRs were 11.3 ± 0.9 nm in diameter and 42.3 ± 3.3 nm long, for an aspect ratio of 3.7. The zeta potential was 38 mV at a pH of 6.7.

The LSPR of PEG-coated GNRs had the longitudinal peak at 787 nm, with an 80% width of 70 nm. The ratio of longitudinal to transverse OD was 4.3. GNRs were 11.1 ± 1.2 nm in diameter and 42.9 ± 3.0 nm long, for an aspect ratio of 3.9. The zeta potential was 3.5 mV at a pH of 7.1. Additional information on the 40 nm GNR used here can be found at the Nanohybrids website.

CTAB-coated GNRs of ~100 nm length were custom made in accordance with the protocol described in^76^. Their parameters were: length 99.7 nm, diameter 35.0 nm, (aspect ratio 2.8), and LSPR peak at 752 nm.

### Numerical simulation of GNR absorption and scattering spectra

FDTD simulations were performed (Lumerical Solutions, Inc. - version 8.19.1466) using a partially rounded cylinder model for nanorods 42.9 nm long by 11.1 nm wide (~40 nm GNR) and 99.7 nm long by 35 nm wide (~100 nm GNR). The spheres used to model the ends of the rod had a radius of 5.55 nm and 30 nm (for 40 nm and 100 nm correspondingly) and were positioned so that the cross-sectional area matched the circular cross section of the cylinder. The rod was then shaped using a dielectric material ring matching the background index. The permittivity of gold was modeled by the Johnson and Christy gold dielectric function^77^. A simulation box with perfectly matched layer (PML) boundaries was used with a mesh size of 0.5 nm and 2 nm (for 40 nm and 100 nm correspondingly), with an override mesh refinement of 1 nm directly over the nanoparticle to ensure convergence. A background refractive index of 1.33 was chosen to simulate an aqueous environment. A broadband total-field scatter-field with a range of 400–1,000 nm with linear 1 nm spacing was used as the illumination source.

To compare computed GNR optical spectra with experimentally measured ones (see Fig. 3), simulated data were homogenously broadened to the linewidth of the experimental data while conserving area, consistent with an ensemble distribution of particle sizes.

### Cell culture and internalizing GNRs into cells

Primary human umbilical vein endothelial cells (HUVECs; C2519A; Lonza) were cultured in EGM-2 medium (CC-3162; Lonza) and maintained at 37 °C in an incubator with 5% CO_2_. HUVECs were used until passages 6–7 and detached by trypsin-EDTA (0.05% trypsin, GIBCO, Grand Island, New York). To internalize GNRs into HUVEC, we used the following procedure. A PEG-coated GNR suspension of 1 mL, OD 100 was diluted by Phosphate Buffered Saline (PBS (Corning) to achieve OD 10, filtered through a 0.45 μm pore-size syringe filter (Whatman) and diluted with cell growth medium to 3 mL. This solution (GNR concentration OD~8.3) was then added to a flask with HUVEC culture to total 12 mL of liquid. After incubating cells with GNRs at 37 °C for 24 hours, they were harvested. Cells were rinsed with Dulbecco’s phosphate-buffered saline (DPBS) solution, then 4 mL of trypsin-EDTA was added. Cells were then rinsed again with DPBS, and growth medium was added before centrifugation. In total, cells were washed three times to remove extracellular GNRs. Pictures of vials with HUVEC control and HUVEC-GNR (Fig. 4d) were taken after centrifugation. Then, cells were fixed at room temperature in 4% formaldehyde (3 ml) for 20 min and used for PA and UV-VIS measurements.

Microscopic images of HUVEC cells in a cuvette of PA spectrophotometer were captured by an optical microscope Olympus Eclipse Ti-U.

## Electronic supplementary material

SUPPLEMENTARY INFORMATION
