# Supplementary material for: Molecular fingerprinting of nanoparticles in complex media with non-contact photoacoustics: beyond the light scattering limit
Source: Sci Rep. 2018 Sep 26;8:14425. doi: 10.1038/s41598-018-32580-2 (PMC6158233; doi:10.1038/s41598-018-32580-2)
Supplement: Supplementary file 1 — SUPPLEMENTARY INFORMATION [file 41598_2018_32580_MOESM1_ESM.pdf]

Molecular fingerprinting of nanoparticles in complex media with non-contact photoacoustics: beyond the lights scattering limit

***Ivan Pelivanov[1], Elena Petrova[1], Soon Joon Yoon[1], Zhaoxia Qian[2], Kathryn Guye [2], Matthew O'Donnell[1]**

[1]Department of Bioengineering, University of Washington, Seattle, WA 98195, USA

[2]Department of Chemistry, University of Washington, Seattle, WA 98195, USA

SUPPLEMENTARY INFORMATION

Laser excitation and detection of ultrasound signals with non-contact PA spectrophotometry

A schematic of the non-contact PA spectrophotometer is shown in Fig. S1a. Pulsed laser radiation from a diode-pumped nanosecond laser (Laser-Export ltd., model: TiSon-B) tunable in the wavelength range of (700 – 900) nm (see laser calibration curve in Fig. S1b) was directed to a measurement cuvette by means of a broadband mirror. The laser pulse energy at the surface of the sample was controlled with a set of neutral-density filters (calibrated in advance) and measured with an energy meter (Ophir Photonics, model: PE50-DIF-ER-C). The laser could vary the pulse repetition frequency (PRF) from single shot to 4 kHz, reaching the maximum mean power of about 1 W at 1 kHz PRF at 800 nm wavelength. A unique feature of the laser is the ability to switch wavelengths within one ms in any programmable sequence desired. The laser can also be triggered externally with little (~ 2ns) jitter and can support variable PRF. These features are not only attractive for scientific studies, but also for commercial devices. Note that only a few μJ of laser energy (less than one percent of its maximum value) was used in the current study.

A medium (solution) under study was placed between two non-absorbing quartz plates within a diaphragm 0.1 mm thick and 6 mm in diameter. The laser beam diameter at the surface of the sample was approximately 0.5 mm.

To homogeneously probe the medium and average possible sample heterogeneities over the entire medium volume and reduce sedimentation of cells, the measurement cuvette was placed on an XY translation stage moved with a linear speed of up to 100 mm/s (80 mm/s used). Translation was positionally synchronized with laser firing, providing a trigger signal to the laser for every 0.5 mm of translation. One hundred positions, and therefore laser shots, were used for one XY-scan of the sample; scans were then repeated fifty (50) times resulting in five thousand (5000) laser shots for every wavelength in the (700 – 900) nm range, which took around 30

seconds per wavelength point. Spectroscopic measurements were performed with a step of 10 nm, resulting in 21 points in total over a complete experiment period of 10.5 minutes.

Due to light absorption in the sample, laser pulses were partially absorbed; the absorbed laser energy was then converted to transient heating of the medium which resulted in the excitation of ultrasound transients (photoacoustically-generated ultrasound (US) signals, or PA signals). “Narrow beam” irradiation conditions were applied to strongly reduce the dependence of the PA signal amplitude on laser fluence [1, 2]. Generated US signals propagating back into the upper quartz plate were recorded with a fiber-optic Sagnac interferometer [3-5] from the top surface of the plate with a reflective coating tuned to 1550 nm (to provide efficient reflection of the probe continuous wave (CW) radiation from the fiber-optic Sagnac interferometer). Transmission through the coating in the (700 – 900) nm wavelength range was also taken into account.

The double differential fiber-optic Sagnac interferometer is a key part of the proposed PA method. Its operating principle was described in detail in Refs. [3-5]. Briefly, this interferometer is different from all other types of two-beam interferometers that split the original beam in two in space, where one beam is the reference and the other is directed to the sample (see Fig S1c).

There are several problems with conventional designs which make it difficult to achieve high sensitivity and robust operation in real, noisy (non-laboratory) conditions. First, conventional designs require a highly coherent source very sensitive to sample surface quality; second (and the most important) is that any background noise applied to the reference arm affects the interference and can strongly reduce the SNR (signal-to-noise ratio).

The fiber optic Sagnac design, proposed by us a few years ago [3, 4] (see also Fig. S1d), splits the probe beam in time by polarizations. One beam is delayed in the fiber delay line. This delay determines the detection bandwidth. Both beams are reflected from the same point of the sample. On the way back, the propagation paths of the beams are switched with a quarter wave plate located in the detection head of the interferometer. When mixed together, they arrive at exactly the same time and, thus, create nearly 100% interference. Additional noise rejection is reached using a balanced photodetector. The interference signal is directly proportional to acoustic pressure, not to the displacement as for conventional designs. The Sagnac does not have a reference arm and thus does not need any stabilization and feedback, which makes it nearly insensitive to environmental noise. A very compact, low power, low coherent superluminescent diode (SLD) (Thorlabs, model: SLD1550P-A1) is the source for the interferometer.

The interferometer’s design is very rugged and compact and uses only fiber-optic components. It has been used for laser-ultrasonic non-destructive testing of fiber-reinforced composites and showed great stability and performance for industrial applications [3-8]. As demonstrated in [3], the sensitivity of the detection provided with the interferometer approaches the Johnson-Nyquist thermal noise level (the ultimate limit for all detectors independent of their nature).

Detection head of the interferometer can change the focal distance, numerical aperture of the objective lens and depth of field. In this work, a 78 mm aspheric focusing lens was used resulting in a numerical aperture NA=0.04 with a depth of field of about 5 mm.

The detected signals were digitized with a 14 bit ADC (GaGe, model: Razor Express 14x2 CompuScope) and then processed on a standard PC.

The detected PA signal amplitude is proportional to light absorption coefficient under narrow beam conditions [1, 2]. To obtain the absolute value of light absorption, the system should be calibrated. The calibration procedure consists of measuring the PA signal amplitude in a medium with known absorption. We used a solution of $CuSO_4*5H_2O$ at different concentrations as the reference medium. For one concentration, the UV-VIS spectrum was measured with a conventional spectrophotometer (Thermo Fisher, model: Evolution 300). The PA signal amplitudes measured at different wavelengths in the range of 700-900 nm were compared with that measured with the conventional method and thus the proportionality coefficient was obtained (Figure S1f). The linearity of the PA method was checked over the range 50 mM – 1 M of $CuSO_4*5H_2O$ concentrations (Figure S1e), corresponding to the range of optical absorption coefficients of 1.3 – 25.9 cm^{-1}. Note that linearity of the PA method was investigated in detail in [9] for highly concentrated solutions up to 300 cm^{-1} of optical absorption. Thus, the operating range of concentrations can be highly extended.

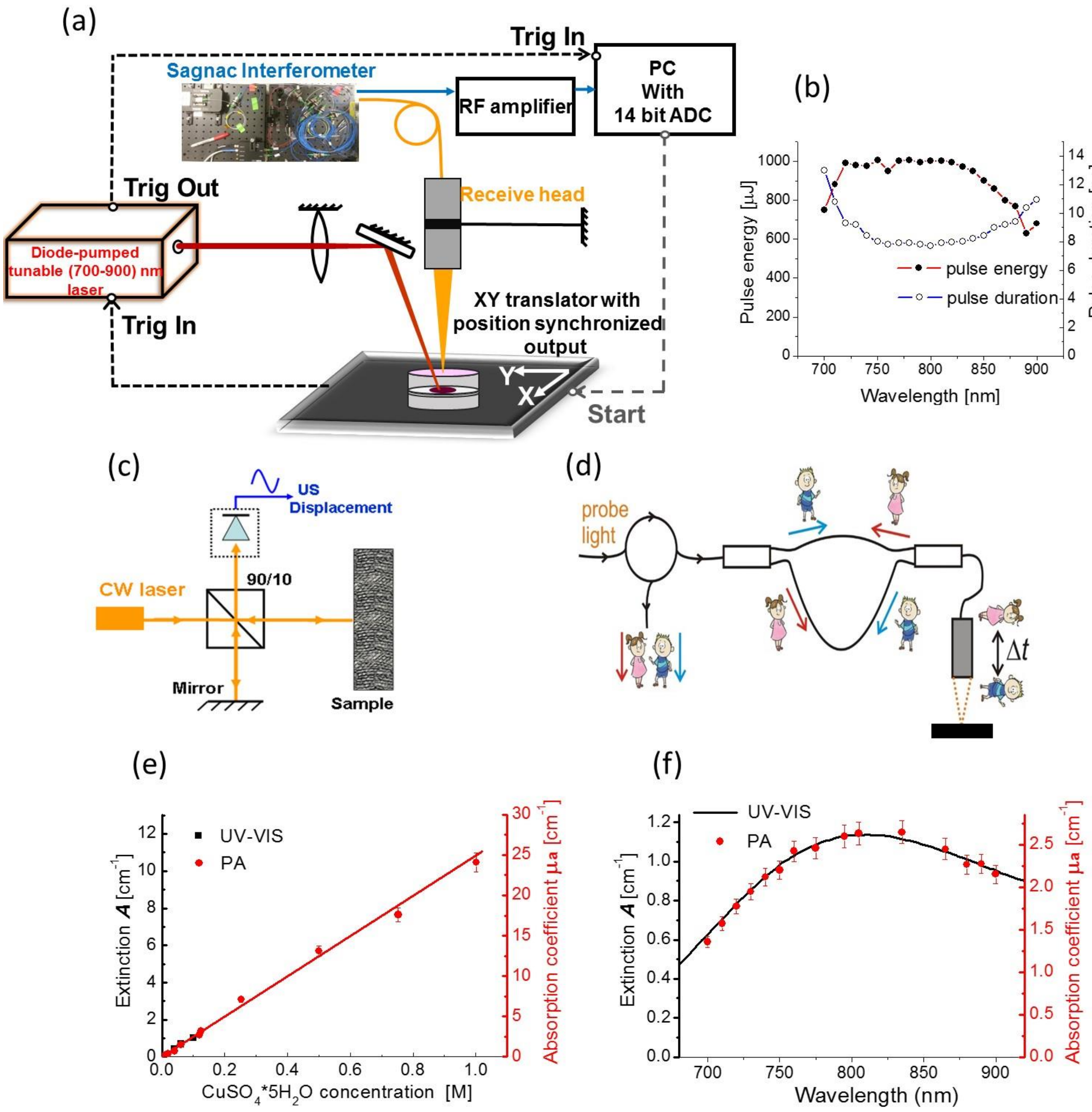

Figure S1. (a) – schematic of the PA spectrophotometer; (b) – calibration curve of the diode-pumped laser tunable in the 700-900 nm wavelength range; (c) - conventional (Michelson, for example) interferometer design; (d) – operating principle of the fiber-optic Sagnac interferometer used in this study; (e) – linearity of the PA method; (f) – calibration curve of the PA spectrophotometer.

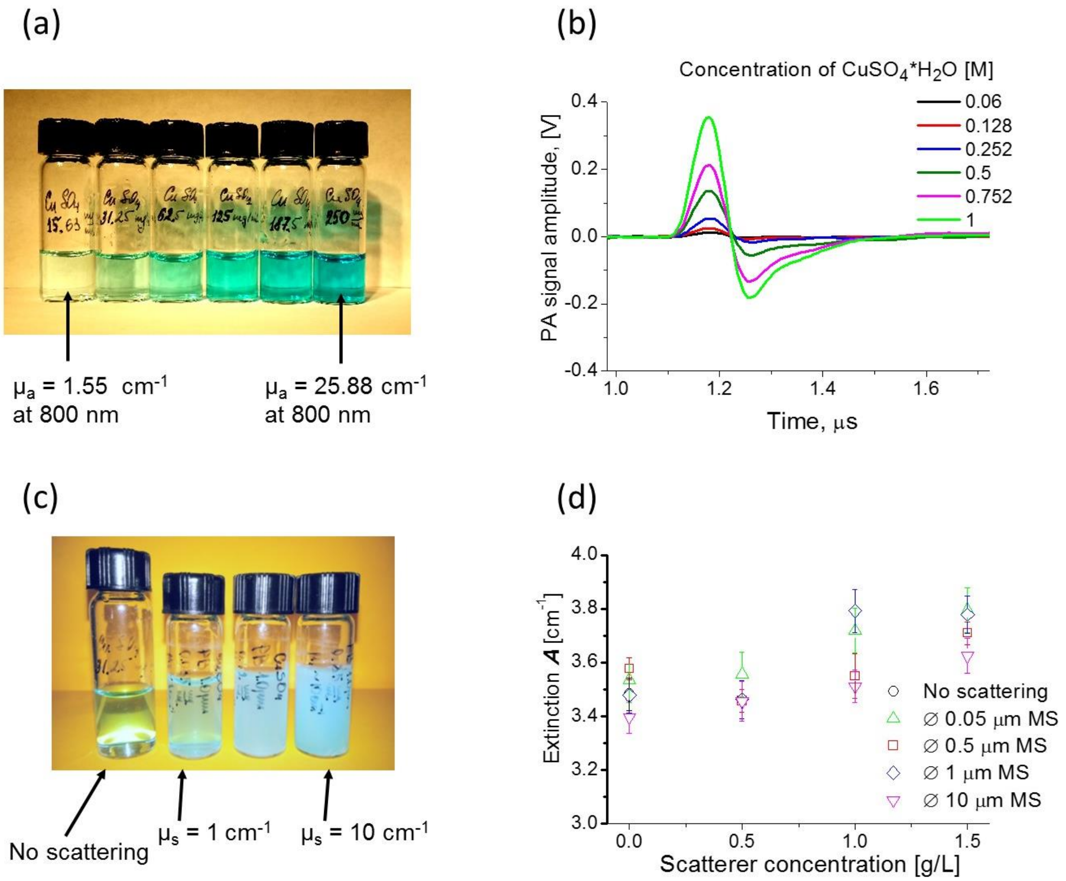

Figure S2. (a) – $CuSO_4*H_2O$ solutions of different concentrations ranging from 60 mM to 1M. (b) – PA signals recorded at these concentrations. (c) - $CuSO_4*H_2O$ solutions with 1 μm diameter polystyrene microspheres taken at the different concentration (from 0.1 to 1.5 g/L); (d) – dependence of the PA signal amplitude on concentration of scatterers (polystyrene microspheres of different diameters) measured at a wavelength of 800 nm.

References

1. Pelivanov, I. M., Barskaya, M. I., Khokhlova, T. D., Podymova, N. B., Karabutov, A. A. Opto-acoustic measurement of the local light absorption coefficient in turbid media: 2. On the possibility of light absorption coefficient measurement in a turbid medium from the amplitude of the opto-acoustic signal. *Quantum Electronics* **39**(9), 835-838 (2009).
2. Pelivanov, I. M., Barskaya, M. I., Khokhlova, T. D., Podymova, N. B., Karabutov, A. A. Opto-acoustic measurement of the local light absorption coefficient in turbid media: 1. Monte-Carlo simulation of laser fluence distribution at the beam axis beneath the surface of a turbid medium. *Quantum Electronics* **39**(9), 831-834 (2009).
3. Pelivanov, I., Buma, T., Xia, J., Wei, C.-W., O'Donnell, M. A new fiber-optic non-contact compact laser-ultrasound scanner for fast NDT&E of aircraft composites. *J. Appl. Phys.* **115**, 113105 (2014).

4. Pelivanov, I., Buma, T., Xia, J., Wei, C.-W., O'Donnell, M. NDT of fiber-reinforced composites with a new fiber-optic pump-probe laser-ultrasound system. *Photoacoustics* **2**, 63-74 (2014).
5. Pelivanov, I., Shtokolov, A., Wei, C.-W., O'Donnell, M. A 1 kHz A-scan rate pump-probe laser-ultrasound system for robust inspection of composites." *IEEE Trans. Ultrason. Ferroelect. Freq. Contr*. **62**, 1696-1703 (2015).
6. Pelivanov, I. & O'Donnell, M. Imaging of porosity in fiber-reinforced composites with a fiber-optic pump-probe laser-ultrasound system. *Composites Part A* **79**, 43-51 (2015).
7. Pelivanov, I., Ambroziński, Ł., O'Donnell, M. Heat damage evaluation in carbon fiber-reinforced composites with a kHz A-scan rate fiber-optic pump-probe laser-ultrasound system. Composites Part A **84**, 417-427 (2015).
8. Pelivanov, I., Ambroziński, Ł., Khomenko, A., Koricho, E. G., Cloud, G. L., Haq, M., O'Donnell, M. High resolution imaging of impacted CFRP composites with a fiber-optic laser-ultrasound scanner. *Photoacoustics* **4**, 55-64 (2016).
9. Filimonova, T. A., Volkov, D. S., Proskurnin, M. A., Pelivanov, I. M. Optoacoustic spectroscopy for real-time monitoring of strongly light-absorbing solutions in applications to analytical chemistry. *Photoacoustics* **1**, 54-61 (2015).
